# Identifying Dietary Triggers Among Individuals with Overweight and Obesity: An Ecological Momentary Assessment Study

**DOI:** 10.3390/nu17030481

**Published:** 2025-01-29

**Authors:** Han Shi Jocelyn Chew, Rakhi Vashishtha, Ruochen Du, Yan Xin Liaw, Ayelet Gneezy

**Affiliations:** 1 Alice Lee Centre for Nursing Studies, Yong Loo Lin School of Medicine, National University of Singapore, Singapore 117597, Singapore; liawyx@nus.edu.sg; 2 Behaviour and Implementation Science Interventions, Yong Loo Lin School of Medicine, National University of Singapore, Singapore 117597, Singapore; rakhi.vashishtha@nus.edu.sg (R.V.); agneezy@ucsd.edu (A.G.); 3 Biostatistics, Yong Loo Lin School of Medicine, National University of Singapore, Singapore 117597, Singapore; ruochen.du@nus.edu.sg; 4 Rady School of Management, University of California, San Diego, CA 117599, USA

**Keywords:** excess adiposity, ecological momentary assessment, dietary triggers, dietary adherence, real-time data collection, weight management

## Abstract

Background/Objectives: Excess adiposity, affecting 43% of the global adult population, is a major contributor to cardiometabolic diseases. Lifestyle behaviours, specifically dietary habits, play a key role in weight management. Real-time assessment methods such as Ecological Momentary Assessment (EMA) provide context-rich data that reduce recall bias and offer insights into dietary triggers and lapses. This study examines dietary triggers among adults with excess adiposity in Singapore using EMA, focusing on factors influencing dietary adherence and lapses. Methods: A total of 250 participants with a BMI ≥ 23 kg/m^2^ were recruited to track dietary habits for one week, at least three times a day, using the Eating Behaviour Lapse Inventory Survey Singapore (eBLISS) embedded within the Eating Trigger Response Inhibition Program (eTRIP© V.1) smartphone app. Logistic regression analysis was used to identify predictors of dietary adherence. Results: Of the 4708 responses, 76.4% of the responses were indicative of adherence to dietary plans. Non-adherence was primarily associated with food accessibility and negative emotions (stress, nervousness, and sadness). Factors such as meals prepared by domestic helpers and self-preparation were significantly associated with adherence. Negative emotions and premenstrual syndrome were identified as significant predictors of dietary lapses. Conclusions: EMA offers valuable insights into dietary behaviours by identifying real-time triggers for dietary lapses. Future interventions can utilise technology-driven approaches to predict and prevent lapses, potentially improving adherence and weight management outcomes.

## 1. Introduction

In 2022, overweight and obesity (henceforth known as excess adiposity) affected 43% of the global adult population [1], translating to a projected increase to 3.29% of the global gross domestic product (GDP) by 2060 [2]. Fuelled by factors such as economic growth, urbanisation, sedentary lifestyles, and dietary changes, excess adiposity increases individuals’ risks of cardiometabolic diseases such as diabetes, hypertension, hyperlipidaemia, stroke, and fatty liver disease, leading to morbidities and mortalities [3,4,5]. While genetics have been shown to play a significant role in excess adiposity, lifestyle behaviours, especially dietary habits, are crucial predictors of body weight and cardiometabolic disease risk [6].

The food frequency questionnaire (FFQ), 24-h diet recall, and dietary records are the most frequently used methods to assess dietary habits but are prone to recall, aggregation, and social desirability biases [7,8]. In the context of dietary habits, research has shown that individuals often hold “self-favouring” perceptions of their eating behaviours, believing that their lapses are less significant or more justified than those of others [9,10,11]. Ecological Momentary Assessment (EMA), defined as the real-time collection of data in participants’ natural environments through repeated sampling over time, [12] has been increasingly popular over the past few decades for tracking various aspects of eating behaviours, including diet choices, meal frequency, and contextual factors. EMA offers significant advantages over traditional assessment techniques by providing immediate and context-rich data, [12] which reduces recall bias and participant burden, resulting in more accurate accounts of daily eating behaviours [13]. In addition to identifying dietary lapses in real time, EMA can detect dietary triggers that traditional methods may miss [14,15,16]. For example, studies utilising EMA have identified common triggers such as negative psychological states like loneliness [17,18], food cravings [19], the presence and variety of palatable foods [14,20], food marketing [21], or the influence of peers and friends [22].

In culturally diverse settings, EMA could potentially reveal how unique cultural factors such as traditional food preferences influence dietary behaviours and body weight. Previous EMA studies conducted in Singapore have found that dietary food patterns and food choices vary substantially among different ethnicities [23,24,25,26,27], potentially explaining the inter-ethnic differences in obesity rates among Singapore residents [28]. However, there is currently limited knowledge on the types of dietary lapse triggers that adults face, especially for those living in multi-ethnic societies, where food is an integral part of the local culture. Therefore, we examine the moment-by-moment specific dietary triggers among adults with excess adiposity in Singapore using EMA.

## 2. Materials and Methods

This study is conducted as part of a larger research project [29]. We recruited 250 individuals with a body mass index (BMI) ≥ 23 kg/m^2^ from the public as well as a specialist obesity management centre. Participants were asked to track their dietary habits over one week through the eating Trigger Response Inhibition Program version 1 (eTRIP© V.1), a smartphone app developed to collect data on individual eating habits, specifically food logs and dietary triggers (i.e., triggers leading to dietary lapses—eating at an unplanned time, an unplanned portion size, or a food item). Based on our previous study, we identified six broad categories of dietary triggers—pre-meal activities, meal companion(s), ease of food accessibility, emotions, physiological conditions, and time of day [30]. These triggers informed the design of the Eating Behaviour Lapse Inventory Survey Singapore (eBLISS), which is embedded in eTRIP© V1 [30]. eBLISS has undergone validation by an expert panel of 10 multidisciplinary healthcare professional team members and is designed to identify real-time dietary triggers experienced by people with excess adiposity through repeated sampling of one’s eating behaviour at least three times daily (breakfast, lunch, and dinner) (manuscript under review). Our findings are reported according to the STROBE checklist (Appendix A).

### 2.1. Daily User Engagement with eTRIP© V.1

At three user-specified mealtimes, predetermined by each participant at the outset of the study and consistently adhered to throughout, the app prompted participants to photograph their meals and respond to a series of questions. Unique dietary lapse triggers were identified as part of the development of the eBLISS and were categorised into seven domains, namely (1) place; (2) emotions; (3) physiological state; (4) eating company; (5) food provider; (6) activity before eating; and (7) number of hours of sleep (manuscript under review). These questions included programmed response options, as well as self-reported yes-or-no questions assessing adherence to one’s diet plan (“Are you adhering to your diet plan?”). If the available responses did not accurately reflect their experience, participants had the option to provide free-text responses for each dietary trigger question. Each check-in required approximately 1–2 min to complete. The participants were prompted three times a day before each of the three meals that locals commonly consume to prevent prompt fatigue. Participants were able to enter their responses for snacks voluntarily.

### 2.2. Data Analysis

All statistical analyses were conducted using the IBM SPSS Statistics 29 [31] with a two-tailed significance level set at 0.05. Free text responses were coded independently by two study team members (HSJC and MS). Responses to multiple-choice questions were dichotomised into two categories, and the frequency and percentage of responses for each category were calculated. A series of univariate generalised estimating equations were conducted to compare responses of adherence and non-adherence for each dietary trigger. Due to the increased risk of type 1 errors arising from the multiple comparisons, Bonferroni adjustment was used such that a *p*-value of 0.001 was needed to indicate significant difference. Relative risks of dietary triggers influencing dietary adherence responses were also calculated.

## 3. Results

Descriptive sociodemographic characteristics of study participants are reported elsewhere [29]. A total of 4708 check-in responses were recorded, representing a 97.7% completion rate of which 1109 and 3599 were identified as non-adherent and adherent to dietary plans, respectively (Table 1). The most common reason for non-adherence was the inability to avoid eating a certain food (55.4%) rather than meal portion and timing. Moreover, 5 out of 48 dietary triggers were significantly different between the two groups but non-significant after Bonferroni adjustment. These includes the place of meal (home/residence); company (alone and with colleagues/peers); ease of obtaining food (self-prepared); and activity prior to eating (travelling).

Adjusting for cluster effect, the age-, sex- and BMI-adjusted relative risk of each dietary trigger contributing to dietary adherence is shown below (Table 2). Only self-preparing food and travelling were significant predictors of dietary adherence responses.

## 4. Discussion

Our findings revealed that 74% of responses were indicative of adherence to diet plans. Consistent with prior research, the inability to resist unintended food types rather than food portion or meal timing was the most common reason for non-adherence [18]. We found significant differences between responses of dietary adherence and non-adherence for dietary triggers, namely place of meal (home/residence); company (alone and with colleagues/peers); ease of obtaining food (self-prepared); and activity before eating (travelling). Instances where individuals prepared their food were 5% more likely to have a dietary adherent response. Instances where individuals travelled before eating were 7% less likely to have a dietary adherent response. Understanding the factors that facilitate adherence, such as avoiding large portion sizes, sticking to planned mealtimes, and avoiding unintended food, provides insights that could be used in the design of future interventions.

Surprisingly, while emotional eating is well recognised to influence dietary non-adherence and weight gain [32], we did not find significant differences between dietary adherence responses for emotions. This could be because individuals have different tendencies to be influenced by different dietary triggers, highlighting that each individual has personalised dietary triggers that require personalised interventions to overcome [33,34]. For example, by using EMA, timely behavioural interventions could increase one’s awareness of an impending event of emotional eating, potentially preventing a dietary lapse event and eventual weight gain [35]. Therefore, when we controlled for a cluster effect arising from different individual response rates, we could have also controlled for such individual differences that statistically dampened the differences at the instance-based level. Nevertheless, we showed that self-preparing food and travelling were significant predictors of dietary adherence that can be generalised. Mills et al. found that people who frequently consume home-cooked meals tend to have a better diet [36]. When travelling, individuals often need to eat outside the home which has been linked to higher energy intake and poorer diet quality [37]. Nago suggested that frequent eating outside the home was positively associated with an increased risk of overweight and obesity [38]. An increasing degree of eating out-of-home was also correlated with a rise in BMI [39]. Furthermore, a study found that pre-meal screen-time activities did not increase subjective appetite and food intake in girls, which is consistent with our results [40].

Contrary to some studies [17,18,23,41,42], our study corroborates that perceived hunger may not be different between adherent and non-adherent individuals, suggesting that non-adherence is likely influenced by other examined factors [43]. More research is warranted to examine the reasons for this discrepancy. Among physiological factors, only premenstrual syndrome (PMS) was negatively associated with dietary adherence. To our knowledge, no other study has previously documented this finding; although, one study suggested that overweight women increase their dietary intake during the pre-menstruation phase [44].

Dietary habits including eating speed, meal frequency, meal timing, diet quantity, and diet quality have been shown to play a crucial role in weight management [45]. By understanding dietary lapse triggers, individuals can improve their eating habits and enhance their weight loss efforts [46]. Forman found that dietary lapse frequency at baseline was inversely correlated with early and overall weight loss [18]. Crochiere identified that momentary increases in urges to deviate from one’s eating plan, cravings, alcohol consumption, and tiredness, as well as a decline in confidence related to meeting dietary goals and planning food intake, were the predictors of dietary lapses [47]. However, adopting and maintaining healthy dietary habits remain challenging as they require a strong level of effortful self-regulation over dietary habits of which episodes are initiated by environmental triggers that could be arrested just-in-time when identified [48]. In other words, even if one is determined to change their dietary habits, they are likely to lapse into maladaptive behaviours before a new healthier dietary habit is established.

### 4.1. Strengths and Limitations

This study had several strengths, including its focus on a multi-ethnic sample and the use of EMA to prevent biases such as recall bias. However, this study was limited by the 1-week timeframe of observation, limiting the potential for more insights to be generated. Like other studies, dietary adherence was also self-reported, limiting the accuracy of our findings due to social desirability bias.

### 4.2. Future Directions

Future research can expand upon our findings and address the noted limitations. Qualitative research could offer meaningful insights into the triggers of lapses and the coping strategies of those who successfully adhere to their diet plan. Investigating the role of other lifestyle factors, such as physical activity, mindfulness, and sleep, in mediating motivation’s influence on dietary adherence is also crucial. Longitudinal studies could shed light on the dynamics of dietary adherence, identifying patterns and predictors of dietary lapses over time. Furthermore, future studies could explore how different triggers identified through EMA impact weight loss outcomes among obese individuals. Understanding the relationship between these triggers and EMA predictions can inform targeted interventions to reduce lapses and promote sustained dietary adherence.

## 5. Conclusions

In sum, we found that non-adherence was primarily associated with food accessibility and negative emotions (stress, nervousness, and sadness). Factors such as meals prepared by domestic helpers and self-preparation were significantly associated with adherence. Negative emotions and premenstrual syndrome were identified as significant predictors of dietary lapses. Our study underscores the potential of technology-driven, tailored, in-the-moment approaches in predicting and preventing dietary lapses. By tracking the dietary triggers and delivering specific coping skills designed to avert lapses, these interventions can significantly enhance dietary adherence. In the future, more sophisticated passive wearable sensors which could measure heart rate during different emotional states would be used to capture changes in the dietary triggers in a continuous manner over time.

## Figures and Tables

**Table 1:** Response count (%) and comparison of each dietary trigger.

Dietary Trigger	Total Number of Responses, Count (%) (N = 4708)	Responses of Non-Adherence to Diet Plan, Count (%) (n = 1109)	Responses of Adherence to Diet Plan, Count (%) (n = 3599)	*p*-Value
**Place**				
Home/residence	2884 (61.3)	628 (56.6)	2256 (62.7)	0.046 *
Work	819 (17.4)	216 (19.5)	603 (16.8)	0.171
Hawker centre/coffeeshop/restaurant/café/food stall	795 (16.9)	204 (18.4)	591 (16.4)	0.728
On the go	78 (1.7)	17 (1.5)	61 (1.7)	0.528
Family’s/friend’s house	57 (1.2)	21 (1.9)	36 (1.0)	0.078
School	45 (1)	13 (1.2)	32 (0.9)	0.707
Event/place of worship/exercise studio/park	30 (0.6)	10 (0.9)	20 (0.6)	0.343
**Emotions**				
Neutral	3288 (69.8)	726 (65.4)	2562 (71.2)	0.175
Happy	1285 (27.3)	330 (29.7)	955 (26.5)	0.149
Stressed/nervous/restless/anxious/worry	425 (9)	125 (11.3)	300 (8.3)	0.495
Bored	307 (6.5)	86 (7.7)	221 (6.1)	0.841
Sad	86 (1.8)	30 (2.7)	56 (1.6)	0.173
Irritated/annoyed/frustrated/angry	19 (0.4)	4 (0.4)	15 (0.4)	0.858
**Physiological state**				
Hungry	1153 (24.5)	281 (25.3)	872 (24.2)	0.775
Tired	827 (17.6)	213 (19.2)	614 (17.1)	0.661
Cold	111 (2.4)	19 (1.7)	92 (2.6)	0.222
Premenstrual syndrome	79 (1.7)	28 (2.5)	51 (1.4)	0.332
Sick	38 (0.8)	9 (0.8)	29 (0.8)	0.566
Full	21 (0.4)	3 (0.3)	18 (0.5)	0.586
Warm	3 (0.1)	1 (0.1)	2 (0.1)	0.399
**Company**				
Alone	2244 (47.7)	482 (43.4)	1762 (48.9)	0.047 *
With family/friends	1692 (35.9)	402 (36.2)	1290 (35.8)	0.999
With colleagues/peers	420 (8.9)	118 (10.6)	302 (8.4)	0.034 *
**Ease of obtaining food**				
Self-prepared	1432 (30.4)	280 (25.2)	1152 (32)	0.007 **
Provided by family	1186 (25.2)	299 (26.9)	887 (24.6)	0.958
Takeaway	934 (19.8)	233 (21)	701 (19.5)	0.129
Self-bought	849 (18)	213 (19.2)	636 (17.7)	0.476
Provided by colleagues/peers	151 (3.2)	40 (3.6)	111 (3.1)	0.451
Provided by friends	114 (2.4)	42 (3.8)	72 (2)	0.09
Provided by domestic helper	42 (0.9)	2 (0.2)	40 (1.1)	0.969
**Activity prior to eating**				
Social media	1163 (24.7)	264 (23.8)	899 (25)	0.489
Working	774 (16.4)	169 (15.2)	605 (16.8)	0.174
Resting/relaxing/watching tv/videos	545 (11.6)	151 (13.6)	394 (10.9)	0.390
Physical activity	518 (11)	112 (10.1)	406 (11.3)	0.238
Eating	305 (6.5)	71 (6.4)	234 (6.5)	0.411
Travelling	274 (5.8)	80 (7.2)	194 (5.4)	0.008 *
Sleeping	267 (5.7)	48 (4.3)	219 (6.1)	0.061
Shopping	227 (4.8)	64 (5.8)	163 (4.5)	0.108
Attending class/reading/studying	158 (3.4)	47 (4.2)	111 (3.1)	0.22
Showering/washing up/preparing to go out	140 (3)	31 (2.8)	109 (3)	0.731
Chatting	104 (2.2)	29 (2.6)	75 (2.1)	0.933
Special occasion	95 (2)	22 (2)	73 (2)	0.745
Preparing a meal	42 (0.9)	10 (0.9)	32 (0.9)	0.289
Gaming	25 (0.5)	3 (0.3)	22 (0.6)	0.342
Caregiving	16 (0.3)	1 (0.1)	15 (0.4)	0.973
Smoking	16 (0.3)	5 (0.5)	11 (0.3)	0.677
Religious activity	7 (0.1)	2 (0.2)	5 (0.1)	0.922
**Adherence (“I planned to:”)**				
I am following my diet plan	3599 (76.4)	0 (0)	3599 (100)	-
Avoid eating this food	615 (13.1)	615 (55.4)	0 (0)	-
Avoid eating this portion	271 (5.8)	271 (24.4)	0 (0)	-
Avoid eating at this time	223 (4.7)	223 (20.1)	0 (0)	-
Sleep	7.0	7.1	7.0	0.458

Note: * *p*-value ≤ 0.05; ** *p*-value ≤ 0.01.

**Table 2:** Relative risk of dietary triggers on dietary adherence response.

Dietary Triggers	Relative Risk	95% Confidence Interval	*p*-Value
**Place**			
Home/residence	1.00	0.97, 1.05	0.721
**Company**			
Alone	1.01	0.97, 1.06	0.574
With colleagues/peers	0.94	0.87, 1.01	0.084
**Ease of obtaining food**			
Self-prepared	1.05	1.01, 1.09	0.04 *
**Activity prior to eating**			
Travelling	0.93	0.87, 0.99	0.03 *

Note: CI = confidence interval; * *p*-value ≤ 0.05.

## Data Availability

The data presented in this study are available upon reasonable request from the corresponding author. The data are not publicly available due to privacy reasons.

## Appendix A

**Table A1:** STROBE Statement—checklist of items that should be included in reports of observational studies.

	Item No	Recommendation	Page No
Title and abstract	1	(a) Indicate the study’s design with a commonly used term in the title or the abstract	1
		(b) Provide in the abstract an informative and balanced summary of what was done and what was found	1
**Introduction**			
Background/rationale	2	Explain the scientific background and rationale for the investigation being reported	1–2
Objectives	3	State specific objectives, including any prespecified hypotheses	2
**Methods**			
Study design	4	Present key elements of study design early in the paper	2
Setting	5	Describe the setting, locations, and relevant dates, including periods of recruitment, exposure, follow-up, and data collection	2–3
Participants	6	(a) *Cohort study*—Give the eligibility criteria, and the sources and methods of selection of participants. Describe methods of follow-up *Case-control study*—Give the eligibility criteria, and the sources and methods of case ascertainment and control selection. Give the rationale for the choice of cases and controls *Cross-sectional study*—Give the eligibility criteria, and the sources and methods of selection of participants	2
		(b) *Cohort study*—For matched studies, give matching criteria and number of exposed and unexposed *Case-control study*—For matched studies, give matching criteria and the number of controls per case	-
Variables	7	Clearly define all outcomes, exposures, predictors, potential confounders, and effect modifiers. Give diagnostic criteria, if applicable	3
Data sources/measurement	8 *	For each variable of interest, give sources of data and details of methods of assessment (measurement). Describe comparability of assessment methods if there is more than one group	3
Bias	9	Describe any efforts to address potential sources of bias	-
Study size	10	Explain how the study size was arrived at	2
Quantitative variables	11	Explain how quantitative variables were handled in the analyses. If applicable, describe which groupings were chosen and why	3
Statistical methods	12	(a) Describe all statistical methods, including those used to control for confounding	3
		(b) Describe any methods used to examine subgroups and interactions	3
		(c) Explain how missing data were addressed	-
		(d) *Cohort study*—If applicable, explain how loss to follow-up was addressed *Case-control study*—If applicable, explain how matching of cases and controls was addressed *Cross-sectional study*—If applicable, describe analytical methods taking account of sampling strategy	-
		(e) Describe any sensitivity analyses	3
**Results**			
Participants	13 *	(a) Report numbers of individuals at each stage of study—eg numbers potentially eligible, examined for eligibility, confirmed eligible, included in the study, completing follow-up, and analysed	3
		(b) Give reasons for non-participation at each stage	3
		(c) Consider use of a flow diagram	-
Descriptive data	14 *	(a) Give characteristics of study participants (eg demographic, clinical, social) and information on exposures and potential confounders	3
		(b) Indicate number of participants with missing data for each variable of interest	3
		(c) *Cohort study*—Summarise follow-up time (eg, average and total amount)	Table 1
Outcome data	15 *	*Cohort study*—Report numbers of outcome events or summary measures over time	3
		*Case-control study*—Report numbers in each exposure category, or summary measures of exposure	
		*Cross-sectional study*—Report numbers of outcome events or summary measures	
Main results	16	(a) Give unadjusted estimates and, if applicable, confounder-adjusted estimates and their precision (eg, 95% confidence interval). Make clear which confounders were adjusted for and why they were included	6
		(b) Report category boundaries when continuous variables were categorized	-
		(c) If relevant, consider translating estimates of relative risk into absolute risk for a meaningful time period	-
Other analyses	17	Report other analyses done—eg analyses of subgroups and interactions, and sensitivity analyses	Table 2
**Discussion**			
Key results	18	Summarise key results with reference to study objectives	7–8
Limitations	19	Discuss limitations of the study, taking into account sources of potential bias or imprecision. Discuss both direction and magnitude of any potential bias	8
Interpretation	20	Give a cautious overall interpretation of results considering objectives, limitations, multiplicity of analyses, results from similar studies, and other relevant evidence	8–9
Generalisability	21	Discuss the generalisability (external validity) of the study results	8
**Other information**			
Funding	22	Give the source of funding and the role of the funders for the present study and, if applicable, for the original study on which the present article is based	9

* Give information separately for cases and controls in case-control studies and, if applicable, for exposed and unexposed groups in cohort and cross-sectional studies. Note: An Explanation and Elaboration article discusses each checklist item and gives methodological background and published examples of transparent reporting. The STROBE checklist is best used in conjunction with this article (freely available on the Web sites of PLoS Medicine at https://journals.plos.org/plosmedicine/, Annals of Internal Medicine at http://www.annals.org/, and Epidemiology at http://www.epidem.com/). Information on the STROBE Initiative is available at www.strobe-statement.org.

## References

[1] World Health Organization (2024). Obesity and Overweight. https://www.who.int/news-room/fact-sheets/detail/obesity-and-overweight#:~:text=In%202022%2C%202.5%20billion%20adults%20%2818%20years%20and,children%20under%20the%20age%20of%205%20were%20overweight.

[2] Obesity W. (2024). Economic Impact of Overweight and Obesity. https://data.worldobesity.org/economic-impact-new/#:~:text=If%20current%20trends%20continue%2C%20using%20data%20from%20161,to%20rise%20to%20%3E%203%25%20of%20GDP%20globally..

[3] Bailey-Davis L., Wood G.C., Benotti P., Cook A., Dove J., Mowery J., Ramasamy A., Iyer N.N., Smolarz B.G., Kumar N. (2022). Impact of Sustained Weight Loss on Cardiometabolic Outcomes. Am. J. Cardiol..

[4] Boushey C., Ard J., Bazzano L., Heymsfield S., Mayer-Davis E., Sabaté J., Snetselaar L., Van Horn L., Schneeman B., English L.K. (2020). USDA Nutrition Evidence Systematic Reviews. Dietary Patterns and All-Cause Mortality: A Systematic Review.

[5] Kinoshita K., Ozato N., Yamaguchi T., Sudo M., Yamashiro Y., Mori K., Ishida M., Katsuragi Y., Sasai H., Yasukawa T. (2022). Association of sedentary behaviour and physical activity with cardiometabolic health in Japanese adults. Sci. Rep..

[6] Weihrauch-Blüher S., Schwarz P., Klusmann J.-H. (2019). Childhood obesity: Increased risk for cardiometabolic disease and cancer in adulthood. Metabolism.

[7] Naska A., Lagiou A., Lagiou P. (2017). Dietary assessment methods in epidemiological research: Current state of the art and future prospects. F1000Research.

[8] Shim J.-S., Oh K., Kim H.C. (2014). Dietary assessment methods in epidemiologic studies. Epidemiol. Health.

[9] Sproesser G., Klusmann V., Schupp H.T., Renner B. (2017). Self-other differences in perceiving why people eat what they eat. Front. Psychol..

[10] Polivy J., Herman C.P., Trottier K., Sidhu R. (2014). Who are you trying to fool: Does weight underreporting by dieters reflect self-protection or self-presentation?. Health Psychol. Rev..

[11] Vartanian L.R., Reily N.M., Spanos S., Herman C.P., Polivy J. (2024). Evidence of a self-serving bias in people’s attributions for their food intake. Appetite.

[12] Shiffman S., Stone A.A., Hufford M.R. (2008). Ecological Momentary Assessment. Annu. Rev. Clin. Psychol..

[13] Schembre S.M., Liao Y., O’Connor S.G., Hingle M.D., Shen S.-E., Hamoy K.G., Huh J., Dunton G.F., Weiss R., A Thomson C. (2018). Mobile Ecological Momentary Diet Assessment Methods for Behavioral Research: Systematic Review. JMIR mHealth uHealth.

[14] Goldstein S.P., Dochat C., Schumacher L.M., Manasse S.M., Crosby R.D., Thomas J.G., Butryn M.L., Forman E.M. (2018). Using ecological momentary assessment to better understand dietary lapse types. Appetite.

[15] Wahl D.R., Villinger K., Blumenschein M., König L.M., Ziesemer K., Sproesser G., Schupp H.T., Renner B. (2020). Why we eat what we eat: Assessing dispositional and in-the-moment eating motives by using ecological momentary assessment. JMIR mHealth and uHealth.

[16] Mason T.B., Smith K.E., Crosby R.D., Engel S.G., Wonderlich S.A. (2021). Examination of momentary maintenance factors and eating disorder behaviors and cognitions using ecological momentary assessment. Eat. Disord..

[17] Goldschmidt A.B., Crosby R.D., Cao L., Engel S.G., Durkin N., Beach H.M., Berg K.C., Wonderlich S.A., Crow S.J., Peterson C.B. (2014). Ecological momentary assessment of eating episodes in obese adults. Psychosom. Med..

[18] Forman E.M., Schumacher L.M., Crosby R., Manasse S.M., Goldstein S.P., Butryn M.L., Wyckoff E.P., Graham Thomas J. (2017). Ecological Momentary Assessment of Dietary Lapses Across Behavioral Weight Loss Treatment: Characteristics, Predictors, and Relationships with Weight Change. Ann. Behav. Med..

[19] Roefs A., Boh B., Spanakis G., Nederkoorn C., Lemmens L., Jansen A. (2019). Food craving in daily life: Comparison of overweight and normal-weight participants with ecological momentary assessment. J. Hum. Nutr. Diet..

[20] Thomas J.G., Doshi S., Crosby R.D., Lowe M.R. (2011). Ecological momentary assessment of obesogenic eating behavior: Combining person-specific and environmental predictors. Obesity.

[21] Boyland E., Spanakis P., O’Reilly C., Christiansen P. (2024). Associations between everyday exposure to food marketing and hunger and food craving in adults: An ecological momentary assessment study. Appetite.

[22] Grenard J.L., Stacy A.W., Shiffman S., Baraldi A.N., MacKinnon D.P., Lockhart G., Kisbu-Sakarya Y., Boyle S., Beleva Y., Koprowski C. (2013). Sweetened drink and snacking cues in adolescents. A study using ecological momentary assessment. Appetite.

[23] Carels R.A., Hoffman J., Collins A., Raber A.C., Cacciapaglia H., O’Brien W.H. (2001). Ecological momentary assessment of temptation and lapse in dieting. Eat. Behav..

[24] Carels R.A., Douglass O.M., Cacciapaglia H.M., O’Brien W.H. (2004). An ecological momentary assessment of relapse crises in dieting. J. Consult. Clin. Psychol..

[25] Patel K.A., Schlundt D.G. (2001). Impact of moods and social context on eating behavior. Appetite.

[26] Schlundt D.G., Sbrocco T., Bell C. (1989). Identification of high-risk situations in a behavioral weight loss program: Application of the relapse prevention model. Int. J. Obes..

[27] Bennett G., Bardon L.A., Gibney E.R. (2022). A comparison of dietary patterns and factors influencing food choice among ethnic groups living in one locality: A systematic review. Nutrients.

[28] Lee Y.S., Biddle S., Chan M.F., Cheng A., Cheong M., Chong Y.S., Foo L.L., Lee C.H., Lim S.C., Ong W.S. (2016). Health promotion board–ministry of health clinical practice guidelines: Obesity. Singap. Med. J..

[29] Chew H.S.J., Chew N.W., Loong S.S.E., Lim S.L., Tam W.S.W., Chin Y.H., Chao A.M., Dimitriadis G.K., Gao Y., So J.B.Y. (2024). Effectiveness of an Artificial Intelligence-Assisted App for Improving Eating Behaviors: Mixed Methods Evaluation. J. Med. Internet Res..

[30] Chew H.S.J., Gao Y., Shabbir A., Lim S.L., Geetha K., Kim G., Chong C.S., Lomanto D., So B.Y.J. (2022). Personal motivation, self-regulation barriers and strategies for weight loss in people with overweight and obesity: A thematic framework analysis. Public Health Nutr..

[31] International Business Machines Corporation (2023). IBM SPSS Statistics for Windows, Version 29.0.2.0.

[32] Chew H.S.J., Lau S.T., Lau Y. (2022). Weight-Loss interventions for improving emotional eating among adults with high body mass index: A systematic review with Meta-Analysis and Meta-Regression. Eur. Eat. Disord. Rev..

[33] Ulusoy-Gezer H.G., Rakıcıoğlu N. (2024). The Future of Obesity Management through Precision Nutrition: Putting the Individual at the Center. Curr. Nutr. Rep..

[34] Gibson A.A., Sainsbury A. (2017). Strategies to Improve Adherence to Dietary Weight Loss Interventions in Research and Real-World Settings. Behav. Sci..

[35] Ahlich E., Goldstein S.P., Thomas J.G. (2023). Characterizing emotional eating: Ecological momentary assessment with person-specific modeling. Appetite.

[36] Mills S., Brown H., Wrieden W., White M., Adams J. (2017). Frequency of eating home cooked meals and potential benefits for diet and health: Cross-sectional analysis of a population-based cohort study. Int. J. Behav. Nutr. Phys. Act..

[37] Landais E., Miotto-Plessis M., Bene C., Maitre d’Hotel E., Truong M.T., Somé J.W., Verger E.O. (2023). Consumption of food away from home in low- and middle-income countries: A systematic scoping review. Nutr. Rev..

[38] Nago E.S., Lachat C.K., Dossa R.A., Kolsteren P.W. (2014). Association of out-of-home eating with anthropometric changes: A systematic review of prospective studies. Crit. Rev. Food Sci. Nutr..

[39] Bhutani S., Schoeller D.A., Walsh M.C., McWilliams C. (2018). Frequency of Eating Out at Both Fast-Food and Sit-Down Restaurants Was Associated with High Body Mass Index in Non-Large Metropolitan Communities in Midwest. Am. J. Health Promot..

[40] Totosy de Zepetnek J.O., Pollard D., Welch J.M., Rossiter M., Faghih S., Bellissimo N. (2017). Pre-meal screen-time activities increase subjective emotions, but not food intake in young girls. Appetite.

[41] Chatterjee A., Gerdes M.W., Martinez S.G. (2020). Identification of risk factors associated with obesity and overweight—A machine learning overview. Sensors.

[42] McKee H.C., Ntoumanis N., Taylor I.M. (2014). An ecological momentary assessment of lapse occurrences in dieters. Ann. Behav. Med..

[43] Stinson E.J., Piaggi P., Votruba S.B., Venti C., Lovato-Morales B., Engel S., Krakoff J., Gluck M.E. (2020). Is dietary nonadherence unique to obesity and weight loss? Results from a randomized clinical trial. Obesity.

[44] Cross G.B., Marley J., Miles H., Willson K. (2001). Changes in nutrient intake during the menstrual cycle of overweight women with premenstrual syndrome. Br. J. Nutr..

[45] Kim J.Y. (2021). Optimal Diet Strategies for Weight Loss and Weight Loss Maintenance. J. Obes. Metab. Syndr..

[46] Spro Chwyl C., Crochiere R.J., Forman E.M. (2023). The role of everyday activities on likelihood of dietary lapse: An ecological momentary assessment study. J. Behav. Med..

[47] Crochiere R.J., Abber S.R., Taylor L.C., Sala M., Schumacher L.M., Goldstein S.P., Forman E.M. (2022). Momentary predictors of dietary lapse from a mobile health weight loss intervention. J. Behav. Med..

[48] Lowe M.R. (2003). Self-regulation of energy intake in the prevention and treatment of obesity: Is it feasible?. Obes. Res..

